# Papillary Thyroid Carcinoma With 5 Unique Point Mutations and Typical Behavior

**DOI:** 10.1210/jcemcr/luaf062

**Published:** 2025-04-11

**Authors:** Aditya Chauhan, Siddhartha Sen, Khalid Amin, Lynn A Burmeister

**Affiliations:** Division of Diabetes, Endocrinology & Metabolism, Department of Medicine, University of Minnesota, Minneapolis, MN 55455, USA; Department of Pathology, University of Minnesota, Minneapolis, MN 55455, USA; Department of Pathology, University of Minnesota, Minneapolis, MN 55455, USA; Division of Diabetes, Endocrinology & Metabolism, Department of Medicine, University of Minnesota, Minneapolis, MN 55455, USA

**Keywords:** papillary thyroid carcinoma, mutation number, *BRAF D594N*, *NRAS Q61H*, *PIK3CA G1007R*, *PTEN*

## Abstract

The frequency and impact of multiple driver mutations have not been extensively explored in papillary thyroid carcinoma (PTC), in which driver mutations are most commonly solitary. We present a case of a 62-year-old female who was found to have a 2.6-cm classical, nonaggressive-appearing PTC. A next-generation sequencing panel assessed the tumor for mutations. Five unique single nucleotide sequence variants, none of which was seen in The Cancer Genome Atlas study on PTC, were found: BRAF D594N, NRAS Q61H, PIK3CA G1007R, PTEN R335*, and PTEN Y225*. We believe that 5 pathogenic variants are the highest reported number for a primary PTC resection specimen to date. The observed typical PTC behavior may be due to a weaker strength of the individual pathogenic variants to drive oncogenic processes. In this case, the high number of genetic alterations did not translate into aggressive histopathology or clinical course.

## Introduction

The molecular pathogenesis of papillary thyroid carcinoma (PTC) most commonly includes solitary driver mutations in either *BRAF, NRAS, HRAS, KRAS* mutations, or fusions of *RET or NTRK* [1, 2]. The Cancer Genome Atlas (TCGA) study on PTC demonstrated strong associations between mutation type and histology, signaling, and differentiation characteristics [1].

Shrestha et al reported a 0.5-cm PTC tumor demonstrating aggressive behavior with extrathyroidal extension and harboring 4 mutations. They suggested the tumor's aggressive behavior might be related to the number of mutations [3]. Little is established about if or how mutation number affects thyroid cancer pathogenesis and behavior.

We present a case of PTC with 5 unique single nucleotide pathogenic variants involving BRAF D594N, NRAS Q61H, PIK3CA G1007R, PTEN R335*, and PTEN Y225* and with typical histopathologic and nonaggressive clinical features.

## Case Presentation

A 62-year-old female with a history significant for smoking, nephrolithiasis, renal cell carcinoma at age 49 years, and total hysterectomy for abnormal Pap smear was incidentally found to have a thyroid nodule on routine examination. Family history was positive for renal cell carcinoma in a brother, thyroid cancer and lymphoma in a sister, melanoma in her father, and breast cancer in a maternal aunt. Her head circumference measured 58 cm. There were a few flesh-colored facial papules and a possible lipoma on the back.

## Diagnostic Assessment

**Figure 1.**
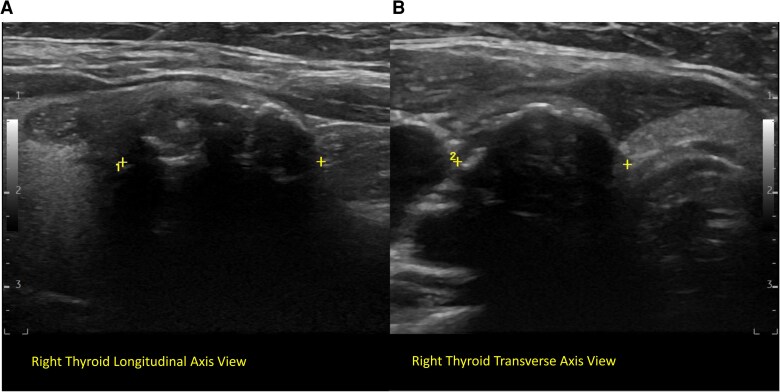
Thyroid and neck ultrasound: longitudinal axis view (A) and transverse axis view (B) images demonstrating a densely calcified right thyroid nodule with marked shadowing and measuring 2.1 × 1.8 cm. This nodule was biopsy-proven papillary thyroid carcinoma.

Thyroid and neck ultrasound showed several TIRADS (TR) 4 and 5 thyroid nodules and no abnormal lymph nodes. The cytology of a densely calcified 2.1-cm TR-4 right nodule (Fig. 1) was reported as PTC.

Laboratory workup revealed TSH 1.52 µIU/mL (reference range: 0.30-4.20 µIU/mL), thyroglobulin 19.10 ng/mL (19.10 µg/L; reference range: 3-40 ng/mL or µg/L), thyroglobulin antibody <0.4 kIU/L (<0.4 IU/mL; reference range: <0.4 kIU/L or IU/mL), calcitonin <0.58 pmol/L (<2.0 pg/mL; reference range: 0.00-1.49 pmol/L or 0.0-5.1 pg/mL), calcium 2.40 mmol/L (9.6 mg/dL; reference range: 2.20-2.55 mmol/L or 8.8-10.2 mg/dL), phosphorus 1.00 mmol/L (3.1 mg/dL; reference range: 0.81-1.45 mmol/L or 2.5-4.5 mg/dL), and PTH 52 ng/L (52 pg/mL; reference range: 15-65 ng/L or 15-65 pg/mL).

For the surgical specimen, the molecular analysis was performed at the Clinical Laboratory Improvement Amendments certified Molecular Diagnostic Laboratory at the University of Minnesota Medical Center. Hybrid capture-based next-generation sequencing (NGS) assay was performed for detection of single nucleotide and insertion-deletion mutations in the coding and splicing regions of the tested genes. Genes tested on the Thyroid Carcinoma NGS Panel (NextSeq) included *AKT1, ALK, BRAF, CTNNB1, GNAS, HRAS, KRAS, NRAS, PIK3CA, PPARG, PTEN, RET, TERT,* and *TP53.*

Genomic DNA was extracted from the fixed tissue specimen, which contained approximately 60% tumor. DNA-enriched sequencing libraries were prepared and then sequenced on an Illumina MiSeq or nextSeq550 instrument. FASTQ files were processed through a custom-developed bioinformatics pipeline to call single nucleotide variants and insertion-deletion variants and to calculate tumor mutation burden (TMB). Variant calling was performed using variant callers VarDict and Mutect2. Variant call files were annotated with GenomOncology software and reviewed for data quality and clinical utility by genomic analysts and board-certified molecular pathologists [4].

The analytic accuracy of the NGS assay is more than 99%. The limit of detection sensitivity of the assay is validated to a minimum threshold of 5% variant allele fraction (VAF); however, variants with variant allele fraction below this threshold may be reported after clinical and data quality review by a molecular pathologist. If the genes included in the panel do not meet the 125× coverage criteria, a manual review of the BAM files is performed, and pathogenic variants may be reported after review by a molecular pathologist.

TMB, or the number of mutations per Mb, was calculated using an internally developed method. The variant call file was generated using VarDict and Mutect2 [5, 6]. Variants were filtered out of the TMB calculation if they were synonymous (annotated by SnpEff, GRCh37.75), ClinVar benign/likely benign (annotated by SnpSift), not located within RefSeq exons, present in an in-house developed mutational hotspot list, present in Exome/Genome database from 1000 genomes or gnomAD, present in an in-house developed common artifact list and had a variant allele frequency ≤0.05 or >0.90. SNPs also had to be called by 1 variant caller to be included in the TMB calculation, whereas Indels required identification by both variant callers to be included [7, 8]. The retained variant number following filtering was then divided by capture size (0.376 Mb) to generate the final TMB value. TMB values are reported numerically based on the established calculation method. TMB classification is reported as low for values <10 mut/MB, high for >16 mut/MB [9, 10].

## Treatment

**Figure 2.**
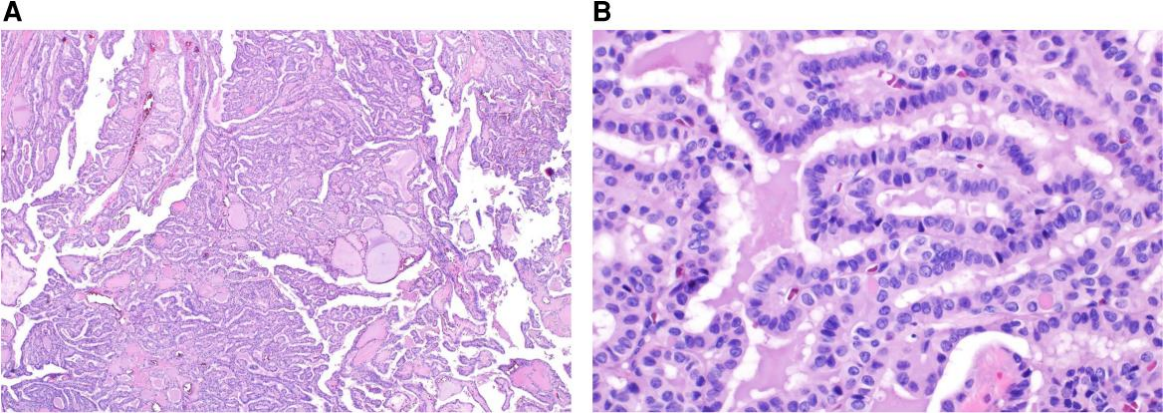
Histopathology of 5-mutation papillary thyroid carcinoma. (A) Low-magnification image of the tumor showing classical papillary architecture of the tumor with a few scattered calcifications (hematoxylin and eosin stain, 4 × magnification). (B) High-magnification image showing cytologic features of the neoplastic cells lining the papillary structures. These features include nuclear enlargement, elongation, grooves, and overlapping as well as characteristic intranuclear pseudo-inclusions seen in the center of the image. (hematoxylin and eosin stain, 40 × magnification).

A total thyroidectomy with the removal of a single delphian lymph node was performed. Histopathology showed a right inferior 2.6 cm PTC, classic/conventional type (Fig. 2). It was encapsulated with negative margins, no extrathyroidal extension, and perineural or lymphatic invasion. Focal angioinvasion (<4 vessels) was noted. Immunohistochemistry of the PTC specimen stained weakly positive for BRAF V600E. The single lymph node was negative for tumor metastasis. Follicular adenoma (1.4 cm) with oncocytic features was also present. The TNM stage was pT2pN0a, MACIS 5.74, and American Thyroid Association recurrence risk was intermediate [11].

TMB was “low” with 7.313 mut/Mb. Pathogenic variants were found for BRAF D594N, NRAS Q61H, PIK3CA G1007R, PTEN R335*, and PTEN Y225*. There was also a *PIK3CA C90Y* variant of unknown significance. Oncology fusion gene panel could not be completed because of a possible DNA quality issue.

## Outcome and Follow-up

Nine-month postoperative thyroglobulin was <0.1 ng/mL (<0.1 µg/L; reference range: 3-40 ng/mL or µg/L), thyroglobulin antibody <0.4 kIU/L (<0.4 IU/mL; reference range: <0.4 kIU/L or IU/mL), TSH 0.93 µIU/mL (reference range: 0.30-4.20 µIU/mL). Neck ultrasound showed no abnormalities at 9 and 20 months after surgery. She declined *PTEN* germline testing.

## Discussion

We describe a case of classical PTC demonstrating 5 missense pathogenic variants, including BRAF D594N, NRAS Q61H, PIK3CA G1007R, PTEN R335*, and PTEN Y225*. We believe that 5 pathogenic variants are the highest reported number for a primary PTC resection specimen to date. The tumor behavior has been typical and nonaggressive on short-term follow-up. Both the number and types of single nucleotide variant found in this tumor are extremely rare and merit discussion.

The TCGA study on PTC-correlated driver mutation with histopathology and *BRAF* or *RAS*-like behavior, emphasizing the mutual exclusivity of the reported single driver mutations [1]. Dual driver mutations, especially when the partner includes *TERT* mutation, have been associated with more aggressive tumor behavior [12-17]. How the number of genetic mutations or specific multiple mutation types could dictate thyroid cancer type or behavior is not established.

None of the single nucleotide variants found in our patient were reported in the TCGA study [1]. Rather, our patient had single nucleotide variants at different DNA loci for the more common driver genes associated with activation of the MAPK or PI3K pathways involved in the pathogenesis of thyroid cancer [2]. It is known that different driver mutations activate the MAPK pathway with different intensities. The intensity of stimulation of the MAPK pathway, including the impact of negative feedback inhibition on the pathway, has been inversely associated with PTC differentiation toward more follicular histologic differentiation [2]. Weaker driver mutations may be associated with thyroid cancer or with benign thyroid neoplasia.

*BRAF V600E* is the most common pathogenic variant in PTC. It is a strong driver mutation, always associated with thyroid cancer, has high kinase activity, and is RAS and dimer independent. *BRAF D594N* is a rare exon 15 single nucleotide variant. It was not described in the TCGA nor in the population of 2961 PTCs screened for exon 15 *BRAF* alterations [18, 19]. *BRAF D594N* is a class 3 mutant, considered kinase-dead, and RAS- and dimer-dependent producing less ERK activation and insufficient feedback to inhibit RAS [20]. Finally, it is of interest that the BRAFV600E immunohistochemical antibody weakly detected the *BRAF D594N* mutant in this case.

NRAS mutation can be found in both thyroid adenoma and thyroid cancer. *NRAS Q61R* is the most common NRAS pathogenic variant in thyroid cancer [21]. In contrast, *NRAS Q61H* was not found in large thyroid cancer populations, including the TCGA or the Genie cohort [19, 21]. RAS activates MAPK with lower intensity than BRAF, signaling through RAF dimers, subject to negative ERK feedback inhibition [2]. *NRAS Q61H* is a poor driver of melanoma formation [22]. Its potency to drive thyroid cancer is unknown.

*PIK3CA and PTEN* mutations are believed to have a driver role in 15 or more cancer types but are generally rare in thyroid cancer [23-25]. *PIK3CA* is more typical of aggressive thyroid cancer types or advanced thyroid cancer, most commonly coexisting with BRAF mutation [25, 26]. *PIK3CA* was found in 3/490 (0.61%) cases in the TCGA, with missense mutation at M1043I and G118D, and deletion at E110. The G1007R found in our case is a gain of function non-canonical sequence variant that has been rarely reported in other cancer types [19].

The frequency of *PTEN* mutation within differentiated thyroid cancer is very low [27]. Our patient had double stop-gain mutation in PTEN, both PTEN R335* and Y225*, resulting in premature stop with protein truncation. Wang et al similarly reported a patient with 4 mutations including 2 *PTEN* mutations [28].

The examination findings, personal and family history of kidney cancer in our case, raise questions of an inherited susceptibility. Cowden syndrome, resulting from a germline *PTEN* mutation, may include kidney cancer and thyroid neoplasm or cancer. Our patient declined *PTEN* germline testing. The allele frequencies of the PTEN R335* and Y225* were 19.5% and 25%, respectively, a combined frequency of approximately 44.5%, close to the heterozygous germline frequency. However, our panel-based testing was performed in tumor tissue only, with no germline or nontumor controls. Therefore, based on tumor-only testing alone, it is not possible to distinguish between somatic and germline mutations.

Our patient had 3 mutations in 2 genes from the AKT pathway and 2 mutations in 2 genes from the MAPK pathway. There were no double mutations in the same gene in the TCGA thyroid cancer population [29]. Double mutations on the same gene or more than 1 mutation in the same pathway may increase the oncogenic impact [29]. In our case, high mutation number alone, even double mutation on the same gene (in our case *PTEN*) did not predict aggressive behavior, presumably because of the mutations being less potent drivers of oncogenic pathways.

TMB is the measurement of the number of mutations that exist within a tumor. It predicts response to immune checkpoint inhibitors in several tumors [9]. The TMB of 7.313 mutation/mB is higher than the average TMB of 0.4 mutation/mB reported in TCGA, both categorized as low [1]. TMB values and interpretation depend on measurement technique, with lower results if assessed by whole exome sequencing (such as used in the TCGA) vs targeted panels (such as used in our case report) [30].

A limitation of this study is that this is a solitary case report with short-term follow-up.

## Learning Points

- To our knowledge, this is the first reported case of classical papillary thyroid carcinoma with 5 genetic mutations, also unique because of the associated unifocal disease, without extrathyroidal invasion, or metastasis, and with no evidence of persistence on short-term follow-up.
- A higher number of pathogenic variants does not always indicate aggressive cancer. Higher mutation number with less potent mutation types might not predict aggressive behavior.
- Both the number and the type of genetic pathogenic variants may interplay in thyroid cancer development and to predict tumor behavior.

## Contributors

All authors made individual contributions to authorship. All authors reviewed and approved the final draft. A.C. and L.B. were involved in conceptualization, investigation, writing-review and editing, visualization. L.B. was also involved in supervision. K.A. was involved in visualization, writing-review and editing. S.S. was involved in investigation, writing-review and editing.

## Data Availability

Data sharing is not applicable to this article as no data sets were generated or analyzed during this study.
